# CGMIM: Automated text-mining of Online Mendelian Inheritance in Man (OMIM) to identify genetically-associated cancers and candidate genes

**DOI:** 10.1186/1471-2105-6-78

**Published:** 2005-03-29

**Authors:** Chris D Bajdik, Byron Kuo, Shawn Rusaw, Steven Jones, Angela Brooks-Wilson

**Affiliations:** 1 Cancer Control Research Program, BC Cancer Agency, 600 West 10^th ^Avenue, Vancouver BC, V5Z 4E6, Canada; 2 Genome Sciences Centre, BC Cancer Agency, 600 West 10^th ^Avenue, Vancouver BC, V5Z 4E6, Canada

## Abstract

**Background:**

Online Mendelian Inheritance in Man (OMIM) is a computerized database of information about genes and heritable traits in human populations, based on information reported in the scientific literature. Our objective was to establish an automated text-mining system for OMIM that will identify genetically-related cancers and cancer-related genes. We developed the computer program CGMIM to search for entries in OMIM that are related to one or more cancer types. We performed manual searches of OMIM to verify the program results.

**Results:**

In the OMIM database on September 30, 2004, CGMIM identified 1943 genes related to cancer. BRCA2 (OMIM *164757), BRAF (OMIM *164757) and CDKN2A (OMIM *600160) were each related to 14 types of cancer. There were 45 genes related to cancer of the esophagus, 121 genes related to cancer of the stomach, and 21 genes related to both. Analysis of CGMIM results indicate that fewer than three gene entries in OMIM should mention both, and the more than seven-fold discrepancy suggests cancers of the esophagus and stomach are more genetically related than current literature suggests.

**Conclusion:**

CGMIM identifies genetically-related cancers and cancer-related genes. In several ways, cancers with shared genetic etiology are anticipated to lead to further etiologic hypotheses and advances regarding environmental agents. CGMIM results are posted monthly and the source code can be obtained free of charge from the BC Cancer Research Centre website .

## Background

Cancers are complex diseases with multiple genetic and environmental factors contributing to their development. The most prominent success stories in cancer genetics to date have involved genes that produce a recognizable pattern of disease within certain rare families. Most cancers, however, are sporadic and appear in people who do not have a clear family history of the disease. These cancers are currently being studied in epidemiological investigations that examine genetics, environmental exposures or both. The studies often compare "cases" or affected individuals to "controls" or unaffected individuals, to determine which group has a higher frequency of a particular gene variant or a greater level of exposure to an environmental agent. The studies require logical hypotheses regarding the genes to be tested and clear criteria for case definition. Cases may be defined as people who have any of several types of cancer, if those types are related. For example, epidemiologic studies of BRCA1 mutation carriers might benefit from information collected about both breast and ovarian cancer cases. But what genes are associated with a group of cancers, and what cancers are associated with a particular gene? The answers can be found in literature regarding cancer genetics, microbiology, clinical medicine, epidemiology and other sciences. More than 1% of all human genes are associated with cancer [1] and information about the association between genes and cancer changes constantly.

Online Mendelian Inheritance in Man (OMIM; ) is a computerized database of information about genes and heritable traits in human populations. The database was created by Victor McKusick at Johns Hopkins University and is now edited by him and colleagues around the world.[2] We consider it a particularly high-quality data source because it is curated by a knowledgeable team, based on information reported in the scientific literature, and continuously updated. OMIM is maintained on the Internet by the National Center for Biotechnology Information at the US National Institutes of Health.[3] Data mining aims to discover unexpected trends and patterns from large sets of data [4], and the rapid growth of biomedical literature underscores the value of text-mining in particular. Text-mining has been described as a modular process involving document categorization, named entity tagging, fact and information extraction, and collection-wide analysis.[5] In document categorization, a subset of potentially relevant documents is retrieved to increase the efficiency of subsequent steps. Named entity tagging identifies the important entities or objects mentioned in the article, often using a list of synonyms. Fact and information extraction identifies the relationships between entities. Finally, in collection-wide analysis, information extracted from different documents is integrated.

Many research studies aim to explore the association between genes and cancer. The design of these studies requires the identification of appropriate patient groups and candidate genes, and both steps can benefit from effective text-mining of public data sources. OMIM is a high-quality information source and considered a key reference database by the genetics community. Our objective was to establish an automated text-mining system for OMIM that will identify genetically-related cancers and cancer-related genes.

## Implementation

We developed the computer program CGMIM to text-mine OMIM. The software considers 21 major cancer types identified by the National Cancer Institute of Canada [ref [6], page 18, Table 1]. CGMIM recognizes genetically-related cancers by identifying cancer types mentioned in association with a specific gene. For pairs of cancer types, CGMIM generates a table with rows and columns for each cancer type, and cells containing the number of OMIM gene entries that mention an association with those cancers. We refer to this table as the siteXsite matrix. If several OMIM entries mention one type of cancer, and several entries mention another type of cancer, then some entries will mention both types of cancer by chance alone. If the mention of different cancers occurred at random, the expected number of genes (E) in OMIM that mention two specific types of cancer can be estimated as the total number of genes related to cancer, multiplied by the probabilities that an entry mentions each individual cancer type. The latter probabilities are estimated as the proportion of genes in OMIM that are related to each cancer type. Explicitly, if there are N genes related to cancer, G_A _genes related to cancer type A and G_B _genes related to cancer type B, then

E_AB _= (G_A_/N) × (G_B_/N) × N   (1)

where E_AB _is the expected number of genes related to both cancer types A and B. The observed number of genes (O) is the number of OMIM entries that mention both cancer types, and O/E indicates whether the number of genes associated with a pair of cancer sites is different than chance alone would predict. An O/E value of 1.0 indicates the number of entries observed is the number expected by chance. An approximate 95% confidence interval (95% CI) is O/E ± (1.96/√E).

Our text-mining algorithm begins by separating paragraphs of an OMIM entry into constituent sentences, and assumes sentences end with a period followed by a space. There are many words and phrases that refer to cancer. A breast cancer might be described as a breast tumor, breast carcinoma or mammary gland neoplasm. A list of synonyms for each cancer type was developed using the International Classification of Disease for Oncology (ICD-O) [7] and augmented by familiar lay terminology. Other variation occurs as the result of English grammar. Breast cancer might be referred to as cancer of the breast, and several cancers might be referred to in a list (e.g., "cancer of the ovary, breast, and skin"). The algorithm identified OMIM entries for each type of cancer by finding sentences that included both a site synonym and a cancer synonym. For phrases in the synonym list, CGMIM searched for sentences containing all of the individual words.

"Stemming" was used to remove capitalization and common suffixes from words, and thereby changes similar words to identical word fragments. The process is best demonstrated with an example.

### Unstemmed

Large-cell lymphomas comprise approximately 25% of all non-Hodgkin lymphomas in children and young adults, and approximately one-third of these tumors have a t(2;5)(p23;q35) translocation.

### Stemmed

larg-cell lymphoma compris approxim 25% of all non-hodgkin lymphoma in children and young adult, and approxim on-third of these tumor have a t(2;5)(p23;q35) transloc

We used an established algorithm ("Porter's algorithm") to perform the stemming.[8]

Our list of synonyms was stemmed and then compared to the stemmed sentences in OMIM. An OMIM entry may contain alternative entry names, mapping information, a text summary, references to key publications, examples of known allelic variants, and a clinical synopsis of the corresponding phenotype. Some of these fields are subjective, such as the examples of allelic variants, and we restricted our search to the text summary.

Finally, not all OMIM entries refer to specific genes. Some entries refer to heritable traits for which no gene has been identified. In addition, more than one OMIM entry can refer to the same gene. This typically occurs when the entry for a trait is linked to a gene that was previously identified and described in a separate OMIM entry. Because OMIM is dynamically organized and updated, this type of multiple referencing is unavoidable. To restrict searches to only the OMIM entries for genes, CGMIM compares each entry name and alternative names with a list of gene names assigned by the Human Genome Organization (HUGO; ).

We performed manual searches of the OMIM database to identify the strengths and weakness of the computerized search method, and to iteratively modify the software. This involved selecting a sample of OMIM entries and reading through the text to determine whether the entries referred to a cancer, or if entries were identified by CGMIM where, in reality, there was no true cancer reference. We also reviewed the entries to identify sentences that referred to cancer, but for which evidence indicated there was no association. (E.g., "An early study showed the gene was not related to breast cancer.") While an OMIM entry might include a sentence of that sort, another sentence in the entry might cite evidence supporting the association. (E.g., "A subsequent study showed the gene was related to breast cancer.") Despite the negative statement, this example OMIM entry mentions evidence supporting the association and hence would be included when tallying entries associated with the cancer.

CGMIM was written in the Perl computer language and implemented on a Linux workstation. OMIM is updated daily and we created static copies of the database to provide a stable reference for search evaluation. The copies of OMIM used to develop CGMIM were downloaded between March and October of 2003, and each copy contained more than 14,000 entries.

## Results and discussion

In the OMIM database on September 30, 2004, CGMIM identified 1943 genes related to cancer. BRCA2 (OMIM *164757), BRAF (OMIM *164757) and CDKN2A (OMIM *600160) were each related to 14 types of cancer. The OMIM entries for all three genes mention leukemia, melanoma, breast cancer, colorectal cancer, pancreatic cancer, stomach cancer, ovarian cancer and prostate cancer. The entry for BRCA2 also mentions cancer of the brain, larynx, cervix, uterus, thyroid and kidney. The entry for BRAF also mentions lymphoma and cancer of the lung, bladder, testes, cervix and uterus. The entry for CDKN2A also mentions lymphoma and cancer of the lung, bladder, brain, esophagus and kidney. Each gene defines a large group of related cancers.

**Figure 1:**
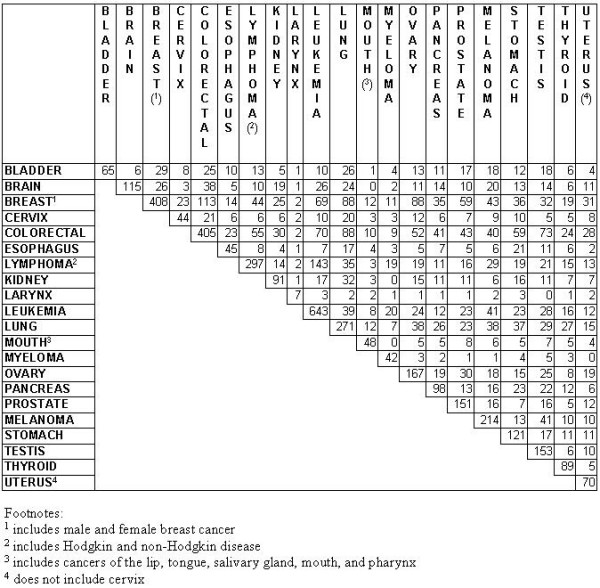
A siteXsite matrix for 21 major cancer types as reported by the National Cancer Institute of Canada. Matrix cells indicate the number of genes related to cancers named in the row and column labels. Cell entries are based on cancers mentioned in Online Mendelian Inheritance in Man (OMIM; ) searched September 30, 2004.

The numbers of genes associated with each pair of cancer types are summarized in the siteXsite matrix (Figure 1). Diagonal cells in the matrix contain the total numbers of genes identified for each cancer type; off-diagonal cells are the numbers of genes identified by both the row and the column titles. For example, there were 45 genes related to cancer of the esophagus, 121 genes related to cancer of the stomach, and 21 genes related to both. The cancer mentioned by the greatest number of OMIM entries was leukemia, and the greatest number of OMIM gene entries that mention a combination of two cancers was 143 for lymphoma and leukemia. For some pairs of cancer sites, no genes were identified.

The numbers in the off-diagonal cells depend on the number of genes related to the individual cancers. Based on the number of OMIM entries that mention leukemia and lymphoma individually, the number expected to mention both is 98.3 and the ratio of the observed and expected values is 1.5 (95% CI 1.3–1.7). (In equation (1), G_LEUKEMIA _= 643, G_LYMPHOMA _= 297 and N = 1943.) This indicates there are 50% more genes related to both cancers than would be expected by chance. Table 1 provides a list of 20 pairs of cancer types where the ratio of the observed and expected number of genes in the siteXsite matrix is greatest. The table indicates that fewer than three genes in OMIM should mention both cancer of the esophagus and cancer of the stomach by chance, but 21 entries mention both cancers. This more than seven-fold discrepancy suggests that cancers of the esophagus and stomach might be more related than current literature suggests. Similar conclusions might be made for the other pairs of cancer types in Table 1.

We randomly selected 25 genes related to cancer and manually reviewed text of the corresponding OMIM entries. All of the entries correctly mention one or more types of cancer, but for 20% of those entries, one of the cancers was only mentioned in the context of evidence suggesting no association.

CGMIM can assist in designing effective studies of genetically-related cancers. CGMIM uses a high-quality database of genetic information to produce a summary of gene and cancer associations. A group of cancer types might be related by physical proximity in the body (e.g., prostate and bladder cancer), a shared physiologic function (e.g., cancers involving the digestive tract), a common exposure (e.g., cancers caused by air pollution) or a common genetic characteristic (e.g., cancers in tissues that express BRCA1). The identification of such groups becomes more difficult and time-consuming as the literature about genes and cancer expands, and efficient text-mining tools have increasing value.

In several ways, groups of cancers that have shared genetic factors are anticipated to lead to further etiologic hypotheses and advances regarding environmental agents. First, grouping cancers will be especially useful if a group combines several cancers that are rare and difficult to study individually. Second, knowledge of genetic pathways might suggest an environmental factor associated with all of the cancers. For example, a grouping defined by a vitamin receptor gene would suggest vitamin intake as a possible environmental agent in the etiology of all of the cancers. Third, CGMIM will allow us to design studies that might extend gene-cancer associations to include cancers at other sites. The groups can also be used to identify cancers that should be considered together in a definition of family history, and in selection of genetic tests that might be adopted for high-risk families. During development of CGMIM, we observed changes in OMIM and the cancer groups that it produced from one week to another. This illustrates the need for a tool that can routinely perform the analysis, as opposed to a set of results based on the OMIM contents from a particular day.

OMIM is based on published material from the scientific literature. The number of genes identified by our program does not necessarily indicate the relatedness of two or more cancer types, but rather what is known about those cancers. This reflects what research has been funded, performed and published. There is more funding for certain types of cancer, there are more journals that address certain types of cancer, and there are more people studying certain types of cancer. Published information reflects our knowledge base and the scientific literature is hence a valid basis for identifying cancer groups and genes for further study. In some cases, evidence about an association was based on studies of cell lines or non-human organisms. In other cases, evidence was based on anecdotal observations in a small number of people. Some associations were based on several independent studies that each involved hundreds of patients.

There are sentences in OMIM that contain phrases such as "is not related to breast cancer". We could not create an algorithm that recognized all negative references without overlooking positive valid ones. Some OMIM entries report both negative and positive evidence of an association. These "mixed" entries are tallied as positive reports by CGMIM, consistent with our interest in positive associations. Other sentences in OMIM describe evidence of gene expression in both cancerous and normal tissue. E.g., "... has been shown to be expressed in breast cancer cells and prostate cells". The sentences are incorrectly interpreted as mentions of prostate cancer. Manual review of OMIM indicated that a minority of apparent associations (about 20%) between a gene and specific type of cancer were the result of negative evidence and are thus "false-positive" text-mining associations. We suggest that a manual review of OMIM associations always precede subsequent study design and analysis. We assume the excess 20% is included in every cell of the siteXsite matrix. Thus expected values also include the 20% excess, and the O/E ratios are not affected.

Other databases might be used as the basis for assessing scientific knowledge regarding genetic cancer groupings, but OMIM offers several advantages. OMIM is based on all publications in the PubMed database that are related to a specific human gene or trait. Results based on mining all of PubMed would be of interest, but would involve a much larger volume of literature and lack the expert review that is characteristic of OMIM. More specialized cancer groupings also might be created using computerized conference proceedings or journal contents. Likewise, a list of synonyms might be determined from other sources such as the UMLS (Unified Medical Language System) Specialist Lexicon of the National Cancer Institute. We used ICD-O terminology because it is the basis for most scientific writing on cancer.

This project used resources that have been developed by the US National Institutes of Health and Human Genome Project.[3] Our approach is exhaustive of the information reported in OMIM, will produce a computer algorithm for near-automatic updating of the review, and has the potential to be extended to other computerized databases. We will use CGMIM along with other criteria to guide the design of studies of genes and environment in cancer etiology.

## Conclusion

CGMIM uses an expert database of genetic information to determine a summary of gene and cancer associations. The software identifies genes that are associated with a particular type of cancer, groups of cancers that share a common genetic association, and pairs of cancer types where there are more related genes than expected by chance.

## Availability and requirements

• **Project name: **CGMIM

• **Project home page: **

• **Operating system: **The source code for CGMIM can be downloaded from the CGMIM homepage and run under Linux.

• **Programming language: **The source code for CGMIM can be downloaded from the CGMIM homepage and is written in Perl.

## Abbreviations

OMIM is Online Mendelian Inheritance in Man; HUGO is the Human Genome Organisation; ICD-O is the International Classification of Disease for Oncology

## Authors' contributions

The software was developed by BK and SR under the direction of SJ and CDB. The website and manuscript were created by CDB and AB. Funding for the project was obtained by CDB, AB and SJ.

## Figures and Tables

**Table 1:** The twenty pairs of cancer types with the highest ratio of observed (O) to expected (E) number of associated genes. The O/E ratio and a 95% confidence interval (95%CI) are provided. Results are based on cancers mentioned in Online Mendelian Inheritance in Man (OMIM; ) searched September 30, 2004.

Pair of Cancer Types	Number of Genes Related to Both		O/E Ratio and 95%CI
	Observed (O)	Expected (E)	
cervix – larynx	2	0.16	12.6 ± 4.9
larynx – mouth	2	0.17	11.6 ± 4.8
larynx – uterus	2	0.25	7.9 ± 3.9
esophagus – stomach	21	2.80	7.5 ± 1.2
larynx – stomach	3	0.44	6.9 ± 3.0
			
bladder – esophagus	10	1.51	6.6 ± 1.6
larynx – myeloma	1	0.15	6.6 ± 5.1
larynx – esophagus	1	0.16	6.2 ± 4.9
cervix – esophagus	6	1.02	5.9 ± 1.9
bladder – cervix	8	1.47	5.4 ± 1.6
			
cervix – uterus	8	1.59	5.1 ± 1.6
larynx – bladder	1	0.23	4.3 ± 4.1
pancreas – stomach	23	6.10	3.8 ± 0.8
cervix – stomach	10	2.74	3.7 ± 1.2
esophagus – mouth	4	1.11	3.6 ± 1.9
			
brain – kidney	19	5.39	3.5 ± 0.8
bladder – testis	18	5.12	3.5 ± 0.9
bladder – prostate	17	5.05	3.4 ± 0.9
bladder – pancreas	11	3.28	3.4 ± 1.1
cervix – lung	20	6.14	3.3 ± 0.8
